# Rapid and Accurate Quantification Detection of BHT in Edible Oils Using Raman Spectroscopy Combined with Chemometric Models

**DOI:** 10.3390/foods15040730

**Published:** 2026-02-15

**Authors:** Congli Mei, Shuai Lu, Xiaolin Zhou, Fanzhen Meng, Hui Jiang

**Affiliations:** 1 College of Electrical Engineering, Zhejiang University of Water Resources and Electric Power, Hangzhou 310048, China; meicl@zjweu.edu.cn; 2 School of Electrical and Information Engineering, Jiangsu University, Zhenjiang 212013, China; ls19707330038@163.com (S.L.); 19516735888@163.com (X.Z.); 2212307081@stmail.ujs.edu.cn (F.M.)

**Keywords:** vegetable edible oils, antioxidants, Raman spectroscopy, machine learning, multivariate analysis

## Abstract

The chemical composition of vegetable cooking oils is a key parameter in determining the quality of their products. Antioxidants are widely used in these products to extend their shelf life. In this study, the concentration of butylated hydroxytoluene (BHT) in edible oil was quantitatively determined by Raman spectroscopy combined with chemometrics. Initially, Raman spectra of edible oil samples with varying concentrations of BHT were obtained. Subsequently, three variable selection methods were applied to the pre-processed spectra. Optimised characteristic wavelengths were then used to establish a Radial Basis Function (RBF) neural network and partial least squares (PLS) models. The impact of variable selection on feature wavelengths was evaluated for both models in both independent and combined cases. The results demonstrate that the features identified through multiple variable selection methods correlate highly with the BHT content and can be utilised to develop high-precision detection models. The findings indicate that the PLS model, optimised using competitive adaptive reweighting (CARS), achieved the best prediction performance, with an average $R_{P}^{2}$ of 0.9687, and RMSEP of 3.1211. These results demonstrate the feasibility of using Raman spectroscopy combined with chemometrics for the rapid screening of BHT in edible oils. While the current study focuses on a broad concentration range to validate the method’s linearity, further optimisation is required for trace-level detection to meet strict regulatory limits.

## 1. Introduction

Cooking oil serves a wide array of purposes [1]. It is the primary medium for frying food and provides essential fatty acids that the human body cannot synthesise. During the storage of edible oils, unsaturated fatty acids are the primary targets that interact with oxygen, leading to lipid oxidation and the formation of hydroperoxides and secondary oxidation products, which result in a reduction in the oil’s nutrient content. Specifically, fat-soluble vitamins are degraded during oxidation, and natural antioxidants such as tocopherols in vegetable oils are depleted during autoxidation. These processes contribute to an increase in the acid and peroxide values of oils, both of which are critical indicators of oil quality. To maintain the quality and shelf life of edible oils, the food industry often adds antioxidants [2]. However, unlike natural antioxidants, the safety of some synthetic antioxidants has raised concerns. Studies have indicated that these antioxidants may interfere with the endocrine system, affecting hormone function [3]. Moreover, long-term consumption of high doses of synthetic antioxidants may place an undue burden on the liver and kidneys.

Given that commercial oils are directly consumed in daily diets [4], rigorous quality testing of these products is essential. Traditional testing methods, such as high-performance liquid chromatography (HPLC), gas chromatography–mass spectrometry (GC-MS) [5], and liquid chromatography–mass spectrometry (LC-MS), require expensive equipment, precise procedures, more time, and highly skilled operators [6]. Rapid detection methods, however, offer significant improvements over these traditional techniques by addressing their limitations [7].

In recent years, molecular spectroscopy [8] has garnered significant attention in the detection of food additives. Raman spectroscopy [9], a technique based on the Raman scattering effect [10], allows for the molecular characterisation of samples by analysing the frequency shift in scattered light when a monochromatic laser irradiates a sample [11]. Compared to traditional detection methods, Raman spectroscopy offers several advantages [12]: it requires no complex sample pretreatment, each substance possesses a unique Raman characteristic spectrum [13], and the Raman shift is independent of the incident frequency [14]. These features confer a high degree of objectivity in the characterisation of substances, making Raman spectroscopy a valuable tool in the study of the quality and safety of edible oils.

Machine learning has been widely used for quality and safety inspection of edible oils with good detection capability [15]. A novel machine learning framework was proposed by Wang et al. This study combines Fourier transform near-infrared spectroscopy with an electronic nose and application transformer encoder backbone with a Support Vector Machine regressor (TES) that detects the adulteration level of camellia oil [16], which can help to safeguard the quality of edible oils and the safety of consumers. Jia et al. [17] combined synchronous fluorescence spectroscopy (SFS) with regression algorithms such as Gaussian Process Regression (GPR), Support Vector Machine (SVM), and Back Propagation Neural Network (BP) to quantitatively detect four polycyclic aromatic hydrocarbons (PAHs) in edible oils, namely, benzo(a)pyrene (BaP), benzo(b)fluoranthene (BbF), benzo(a)anthracene (BaA), and chrysene (Chr), achieving excellent quantitative detection performance [18]. Moraes et al. trained a Partial Least Squares Discriminant Analysis (PLS-DA) model by Principal Component Analysis (PCA) after dimensionality reduction, which has good performance in classification of edible oils and oil gels [4]. The above literature proves that machine learning algorithms have excellent results in the field of detection.

Based on the excellent prediction performance of machine learning [19] and the advantages of Raman spectroscopy [20] being fast and non-destructive, many studies in recent years have combined Raman spectroscopy with machine learning to detect the degree of adulteration of pomegranate juice with good performance [21]. This study demonstrates the feasibility of Raman spectroscopy combined with machine learning models for quantitative tasks. In addition, our laboratory has successfully applied Raman spectroscopy [22] with machine learning in qualitative and quantitative studies for contaminant detection in edible oils [23]. However, traditional detection methods (e.g., HPLC) are time-consuming and require complex pretreatment, making them unsuitable for rapid industrial screening. While previous Raman studies have focused on qualitative classification, there is a lack of robust quantitative models capable of extracting weak BHT signals from the complex fluorescent background of diverse oil matrices. This study addresses this gap by systematically evaluating advanced variable selection algorithms to improve the quantitative accuracy of Raman spectroscopy for rapid BHT screening [24]. Furthermore, previous studies have demonstrated the potential of Raman spectroscopy for analysing antioxidants in oil systems, such as the detection of BHA, BHT, and TBHQ [25]. Surface-Enhanced Raman Scattering (SERS) has also been applied for the sensitive detection of BHA [26] and multiple antioxidants in complex oil matrices [27]. These works provide a solid foundation for exploring spectroscopic methods in antioxidant quantification.

The primary objective of this study is to advance the current literature by systematically evaluating the performance of different chemometric strategies for the quantitative detection of BHT in complex oil matrices. The specific goals are: (1) to construct a Raman spectroscopy detection model for acquiring spectra from diverse edible oil samples; (2) to optimise the pre-processed Raman spectra using three different variable selection methods (CARS, BOSS, and IVSO); and (3) to establish and compare detection models using Radial Basis Function (RBF) neural network and Partial Least Squares (PLS) approaches. By contrasting linear and non-linear modelling strategies, this work aims to provide a robust methodological framework for improving the sensitivity and accuracy of Raman-based food additive detection.

## 2. Materials and Methods

### 2.1. Collection and Preparation of Edible Oil Samples

The edible oil samples used in this study were purchased from local supermarkets in Zhenjiang, China. These edible oils were produced under strict supervision and included four types: corn oil, soybean oil, sunflower oil, and peanut oil, all of which were extracted from their respective seeds. The quality indicators of the purchased edible oils complied with the national standard GB 2716-2018 [28]. According to their composition labels, these oils do not contain butylated hydroxytoluene (BHT). To establish a broadly applicable model, it was essential to ensure sample diversity. Therefore, the edible oils selected for this experiment were chosen based on their origins and types. A total of nine distinct commercial edible oil products, covering four botanical types (corn, soybean, sunflower, and peanut) and various brands, were purchased. This diversity was intentionally selected to introduce maximum matrix variability into the dataset, ensuring that the developed model is robust against spectral interferences caused by different fatty acid profiles and backgrounds. Specific information regarding the purchased oils can be found in Table 1. The BHT used in the experiment, a crystalline powder, was sourced from Aladdin Chemical Reagents [29].

BHT is a fat-soluble substance that dissolves readily in fats and oils. To prepare the samples, BHT powder was mixed with cooking oil at a ratio of 1:9 by weight and then sonicated for 15 min at a controlled temperature of 25 °C with a power of 200 W to ensure complete dissolution without inducing oxidation. Although the concentration range (0.05–60 g/kg) extends beyond typical regulatory limits, this wide dynamic range was designed to simulate scenarios of gross adulteration or manufacturing errors and to validate the linearity of the spectroscopic response as a proof-of-concept. The electronic balance used for weighing, model ME55, was manufactured by Mettler Toledo instruments (Shanghai). To avoid human error in preparing samples with small concentration gradients, the initial samples were proportionally diluted, resulting in 14 oil samples with different BHT concentrations. The exact dilution factors were determined gravimetrically to ensure precision. The required mass of the stock solution ($M_{stock}$) added to the blank oil ($M_{oil}$) for each target concentration ($C_{target}$) was calculated using the mass balance equation: $C_{target}=\left(C_{stock}\times M_{stock}\right)÷\left(M_{stock}+M_{oil}\right)$. All weighing operations were performed using the high-precision electronic balance to minimise quantitative errors. These concentrations were 0.05, 0.1, 0.2, 0.4, 0.5, 1, 2, 4, 5, 6, 10, 20, 40, and 60 g/kg. Each concentration level included all nine types of edible oils. Consequently, this study involved 14 concentration gradients, each containing nine oil samples, totaling 126 samples. After preparation, the oil samples were sealed and stored at room temperature in the laboratory for spectroscopic examination.

### 2.2. Spectral Data Acquisition

**Figure 1:**
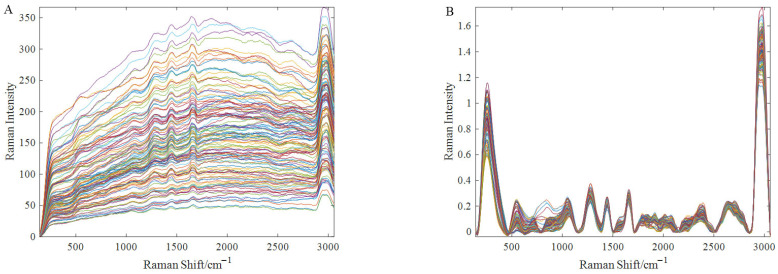
Raw Raman spectra (**A**) and preprocessed Raman spectra (**B**) of edible oil samples. The multiple colored lines represent the individual Raman spectra of the 126 edible oil samples analyzed in this study. These samples encompass nine different commercial brands across four types of vegetable oils (corn, soybean, sunflower, and peanut) and cover 14 BHT concentration gradients ranging from 0.05 to 60 g/kg.

During spectral acquisition, a 5 mm quartz cuvette was used to hold the oil samples. The cuvette was thoroughly cleaned with a strong acid reagent to prevent air bubbles from affecting the spectral data. Raman spectra for each concentration batch were acquired using a QE Pro Raman+ high-sensitivity spectrometer (Ocean Insight, Orlando, FL, USA). A laser source with an excitation wavelength of 532 nm and a power of 10 mW was employed, and the Raman shift range was from 1.592 cm^−1^ to 4538.194 cm^−1^, covering the vibrational modes of most molecules. The spectrometer’s parameter settings were configured as follows: the integration time was 1000 ms, the sliding average width was 5, and the average spectrum of three edible oil samples was considered the raw spectrum of the sample. The spectral data were recorded using the Ocean View software. Each recorded spectrum consisted of 1010 data points; however, spectral regions beyond 3100 cm^−1^ were excluded because this region exhibited significant high-frequency detector noise and artefacts in our experimental setup, which obscured meaningful chemical information. Truncating the spectra at this point improved the signal-to-noise ratio for the fingerprint region, leaving 572 data points. Figure 1A shows the raw Raman spectra of all edible oil samples containing BHT.

### 2.3. Spectral Preprocessing

Raman spectra are influenced by various factors during acquisition, including sample positioning, surface irregularities, laser intensity drift, and fluctuations in ambient temperature, which contribute to significant noise in the acquired spectra. In addition, it can be clearly seen from Figure 1A that the Raman spectra of different types of edible oils are quite different; that is, the nine samples of each gradient have certain differences. As a result, it is essential to preprocess the raw spectra before conducting further analysis. Noise in Raman spectra is typically addressed first through smoothing techniques, such as Savitzky–Golay (SG) filtering, moving average smoothing, and others. To correct for light scattering effects, methods like standard normal variate (SNV) are employed. For spectra with baseline instability, baseline correction is necessary. After careful consideration, performing SG filtering followed by SNV and finally baseline correction was selected as the optimal preprocessing pipeline because it yielded the lowest Root Mean Square Error of Cross-Validation (RMSECV) in preliminary comparative trials, effectively reducing fluorescence background and baseline drift. Figure 1B shows the Raman spectra after preprocessing.

The parameter settings for each preprocessing method are as follows: the window size and polynomial order for SG filtering were 19 and 2, respectively; the smoothing parameter for the iterative adaptive-weighted penalised least squares method (air PLS) was set to 1000, the differential order was 2, the penalty factor was 0.001, the weight update parameter was 0.5, and the maximum number of iterations was 30.

### 2.4. Data Analysis Methods

#### 2.4.1. Feature Selection

All data analyses were performed using MATLAB R2018a (MathWorks, Natick, USA). The variable selection algorithms (CARS, BOSS, IVSO) and PLS models were implemented using the open-source ‘libPLS’ toolbox [30]. Prior to modelling, all spectral data were mean-centred. For CARS, the number of Monte Carlo sampling runs was set to 50. For BOSS, the number of bootstrap runs was set to 1000. For the RBF neural network, the spread parameter was optimised using a grid search method, and the number of hidden neurons was determined by minimising the cross-validation error.

Spectral data is a high-dimensional and complex form of data that is particularly prone to overfitting, making the selection of relevant variables crucial. Numerous methods have proven to effectively and efficiently screen spectral variables and eliminate irrelevant variables.

The Bootstrap Soft Shrinkage (BOSS) first performs Weighted Bootstrap Sampling (WBS) and then combines it with Model Population Analysis (MPA). The method continuously reduces the number of characteristic variables by applying a soft contraction to the regression coefficients of the PLS model. Specifically, BOSS stochastically combines BSS and WBS techniques to generate subsets of variables, which are then PLS-modelled. The absolute values of the regression coefficients from the analysis of the sub-models by MPA were updated with the weight shares of the variables. The so-called soft shrinkage principle means that only the weight share is reduced and not directly eliminated. However, as the number of iterations of the algorithm increases, eventually, the weight share of all variables will be reduced to 0, and thus, the optimal subset will be selected. After multiple runs of the BOSS algorithm, the subset of variables with the smallest root mean square error of cross-validation (RMSECV) is considered the optimal subset. In this study, BOSS is used as a variable selection method, and the number of bootstraps is set to 1000.

Competitive Adaptive Re-weighted Sampling (CARS) is commonly used for feature selection of high-dimensional data such as spectra, where Monte Carlo Sampling (MCS) is used in the sampling session. The subset of variables is modelled by applying PLS to obtain the regression coefficients matrix. Then the subset of variables is dynamically weighted by the Exponential Decay Function (EDF), and finally, according to the Adaptive Re-weighted Sampling (ARS), the importance of the features is adjusted to select the optimal feature subset. After running the CARS algorithm several times, the subset with the smallest root mean square error of cross-validation (RMSECV) is considered the most efficient subset. The number of Monte Carlo sampling runs is set to 50.

IVSO (Iterative Variable Subset Optimisation) is an optimisation algorithm for feature extraction based on partial least squares proposed. It improves the performance and interpretability of the model by selecting and optimising variable subsets iteratively. Firstly, a binary matrix is generated based on weighted bootstrap mean subsampling (WBMS), and the number of variables selected by WBMS is noted as L1. On the basis of the current subset of variables, N partial least squares submodels are created to compute the coefficient matrix B. Summing each row of the normalised matrix B yields the conditional values represented by the vector S, and then using this as a basis for sorting L1, in the sorting order, construct the Sub-model. Based on the desired evaluation metrics, select the appropriate subset of variables from these sub-models. Record the calibrated root mean square error (RMSEC) values and record the size of the subset as L2. Normalise the vector S and calculate the weights. Repeat the above steps until L1 and L2 are satisfied to be of equal size. Finally, output the optimised subset of variables.

#### 2.4.2. Detection Models

Raman spectroscopy is an indirect detection technique that requires an established model to link spectral data with the actual measurements of the samples. The Radial Basis Function (RBF) neural network, known for its accurate local approximation capabilities, is widely used across various fields. The RBF neural network is inspired by the structure of the human brain, specifically simulating the local adjustments and overlapping receptive fields. Its primary function is to transform a non-linearly separable problem in a low-dimensional space into a linearly separable problem in a high-dimensional space. The RBF neural network is particularly effective in handling complex patterns that are difficult to decipher within a system, and it exhibits strong generalisation abilities [31].

Partial Least Squares (PLS) is a regression prediction algorithm particularly suitable for dealing with high-dimensional data like Raman spectra with multicollinearity, i.e., the number of predictor variables is much larger than the number of reference variables. PLS orthogonally decomposes the spectral matrix X (the Characteristic variables) and the reference measurements y (the BHT content) to obtain the latent variables (LVs). In this modelling exercise, the optimal number of LVs was confirmed using five-fold cross-validation. To prevent underfitting as well as overfitting phenomena, the number of LVs with a minimum cross-validated root-mean-square error of minimum (RMSECV) was used as an input parameter to the PLS model.

In this study, an accurate RBF neural network and PLS regression were employed to establish a quantitative model for detecting BHT based on optimised characteristic wavelength variables. The RBF expansion rate in the neural network was fine-tuned using a five-fold cross-validation method combined with a search algorithm.

### 2.5. Evaluation Criteria

The Root Mean Square Error (RMSE) [32] measures the difference between predicted and actual values and is an indicator of the accuracy of a model’s predictions. Mean square error cross-validation (MSECV) evaluates the model’s error during cross-validation, reflecting its generalisation ability. The coefficient of determination ($R^{2}$) represents the proportion of variance in the actual values that the model explains. The closer $R^{2}$ is to 1, the greater the explanatory power of the model. Additionally, the ratio of performance to deviation (RPD), defined as the ratio between the standard deviation of the actual values and the RMSE, is used to assess the accuracy of the prediction model. Generally, it is accepted that, when the RPD falls between 1.4 and 1.8, the model possesses average predictive ability suitable for rough predictions. An RPD between 1.8 and 2 indicates good predictive ability, making the model reliable for more accurate predictions. When the RPD exceeds 2, the model is considered to have excellent predictive ability and can be used for precise predictions.

### 2.6. Software

The programme in this study was run on a Windows 10 operating system equipped with an R5600X model CPU and 16GB of RAM. The study used the well-known Matlab R2018a data software (Mathworks, USA) to run the model.

## 3. Results and Discussion

### 3.1. Division of Sample Set

To ensure a reasonable model construction, this study randomly divided the 126 prepared edible oil samples into a training set and a prediction set. The division ratio was set to 0.7, resulting in 88 samples in the training set and 38 samples in the prediction set. As evidenced in Table 2, the calibration and prediction sets exhibited highly consistent statistical characteristics. Both sets covered the identical concentration range (0.05–60 g/kg), and their standard deviations (17.155 vs. 17.879 g/kg) were comparable, indicating uniform data distribution. Furthermore, both sets contained representative samples from all nine oil types, ensuring that the matrix variability was equally represented in both model training and validation. The specific division results are shown in Table 2.

### 3.2. Results and Comparison of Multivariate Analysis

#### 3.2.1. Results of Variable Selection

**Figure 2:**
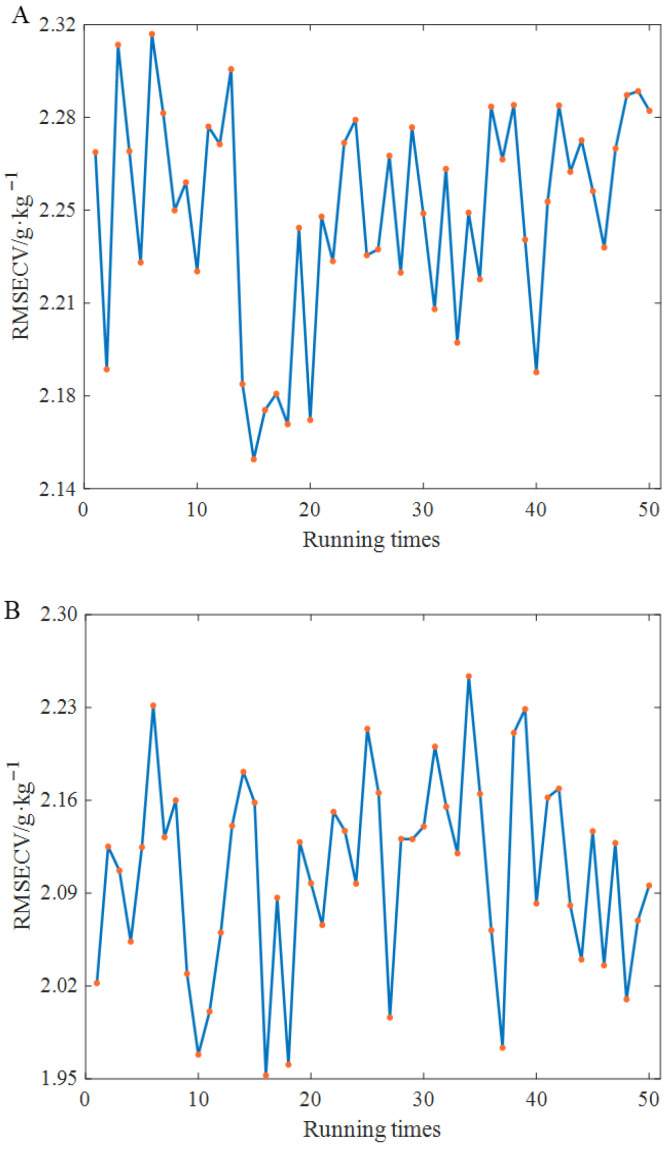

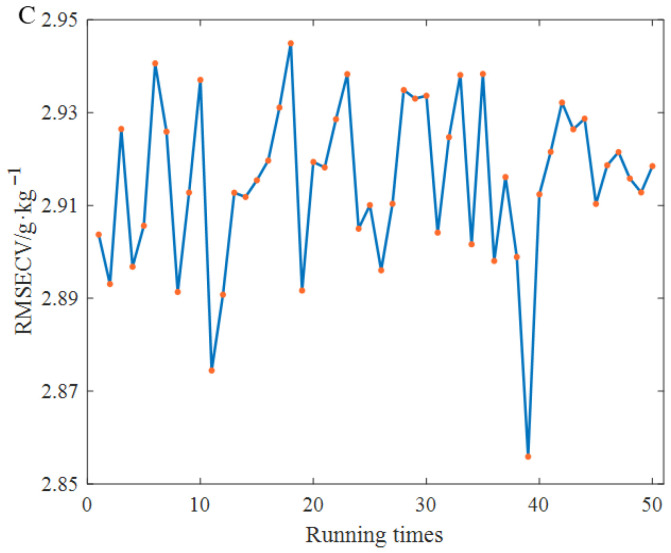
RMSECV variation results and RMSEP variation results of 50 runs with different variable selection methods: (**A**). BOSS; (**B**). CARS; (**C**). IVSO.

Despite preprocessing, external factors still introduce considerable redundant information into the spectra. To address this, several feature selection methods were employed in this study to optimise the feature wavelength variables, specifically BOSS, CARS, and IVSO. Given the inherent randomness in the sampling stages of these methods, each optimisation method was executed 50 times to mitigate the impact of this variability. Figure 2 presents the statistical results of the RMSECV values calculated from the PLS model after running each method 50 times. As shown in Figure 2, all three algorithms exhibit a certain degree of randomness; BOSS, CARS, and IVSO all display slight fluctuations.

#### 3.2.2. Results of PLS Models Based on Different Variable Selection

The characteristic wavelength variables optimised by the three feature selection methods were incorporated into the PLS model, and the evaluation metrics are presented in Table 3. It can be observed that the $R_{P}^{2}$ parameters of all three models exceed 0.9, indicating excellent predictive performance. The Ratio of Performance to Deviation (RPD) was also calculated to further evaluate the predictive ability. As shown in Table 3, the RPD values for the CARS-PLS model exceeded 2.0, indicating that the model possesses good quantitative prediction capabilities. Among these methods, the BOSS-PLS model achieved an average RMSEP of 3.7257 and an average $R_{P}^{2}$ of 0.9553, demonstrating better predictive performance than the IVSO-PLS model. A detailed analysis of the results in Table 3 reveals that the IVSO method selected significantly more optimised characteristic wavelength variables compared to the BOSS method, suggesting that IVSO may have included more redundant features, leading to poorer model performance. Additionally, the CARS-PLS model achieved an average RMSEP of 3.5843 and an $R_{P}^{2}$ of 0.9586, indicating superior predictive performance compared to the BOSS-PLS and IVSO-PLS models. Notably, the CARS-PLS model retained only 10% of the spectral variables, significantly reducing the complexity of the model.

**Figure 3:**
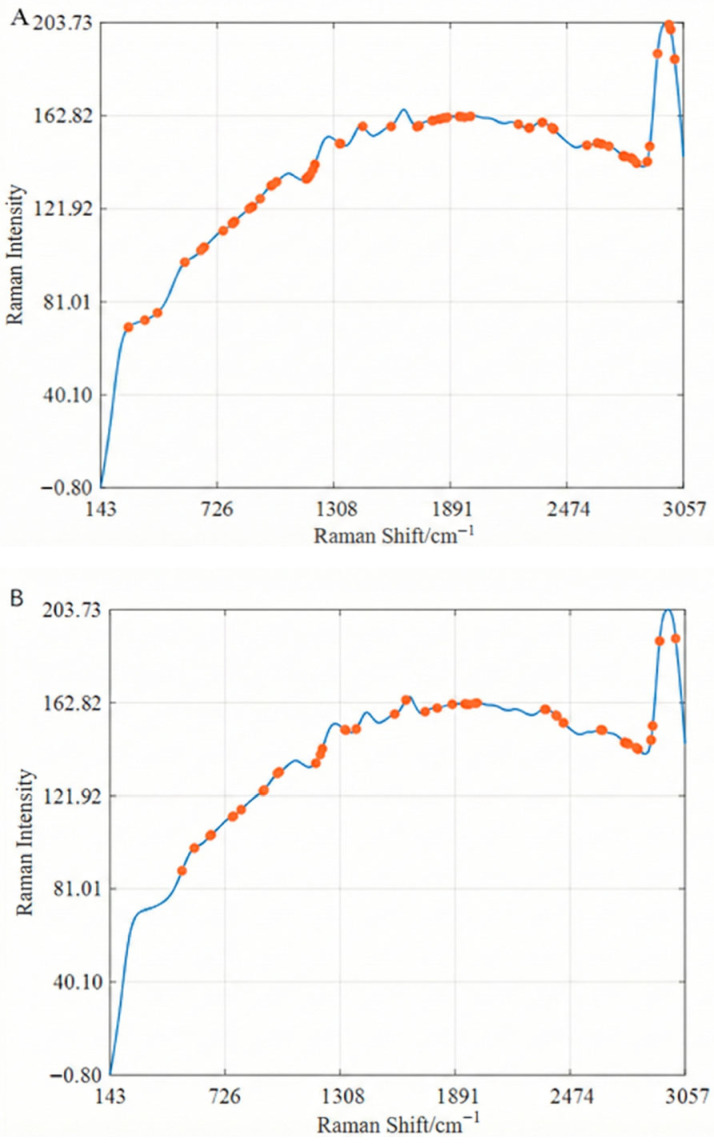

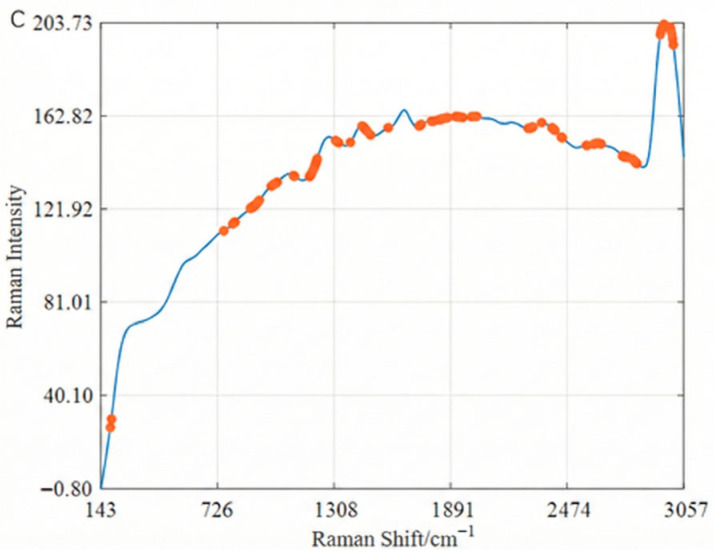
The distribution of characteristic wavelengths optimised by different variable selection methods on the full spectrum: (**A**). BOSS; (**B**). CARS; (**C**). IVSO.

Figure 3 illustrates the distribution of the corresponding feature variables across the full spectrum for the best models optimised by the three feature selection methods. An analysis of Figure 3 reveals that, while there are differences in the number of selected feature wavelengths and their corresponding Raman frequency shift positions, the three methods share some similarities in their selection. For instance, in the bands around 800 cm^−1^, 2800 cm^−1^, and 3000 cm^−1^, all three methods select a relatively higher number of wavelength variables. This observation suggests that the Raman intensity corresponding to the selected characteristic wavelengths is highly correlated with changes in BHT concentration, making them effective in reflecting BHT concentration variations in edible oils.

According to previous studies and standard spectral databases, several Raman bands in the fingerprint region of BHT are associated with vibrational modes of the aromatic ring structure. Rather than relying on a single diagnostic peak, this study focuses on multivariate spectral patterns extracted from the fingerprint region. The variables selected by the CARS, BOSS, and IVSO algorithms correspond to Raman shifts that are strongly related to the characteristic vibrational features of BHT [33]. This multivariate strategy enables effective extraction of BHT-related spectral information from the complex oil matrix, where strong background signals and spectral overlap may obscure individual Raman bands. The Raman peaks at 2954 cm^−1^ correspond to the stretching vibration of the C-H bond on the aromatic ring, while the Raman peak at 2818 cm^−1^ is mainly related to the stretching vibration of the C-H bond in the alkyl group. All of these characteristic wavelength variables are associated with the molecular structure of BHT.

### 3.3. Comparison and Discussion of the Optimal Results of Different Models

Table 4 presents the optimally constructed RBF models based on the three feature selection methods. A comparison of the results in Table 3 and Table 4 reveals that the performance of the nonlinear RBF model is slightly inferior to that of the linear PLS model. Certainly, the significant differences between the RMSEC and RMSEP values of the CARS-RBF, IVSO-RBF, and BOSS-RBF models indicate severe overfitting. In this study, the linear PLS model consistently outperformed the non-linear RBF model, suggesting that the relationship between Raman intensity and BHT concentration is predominantly linear within the tested range. This discrepancy can be attributed to the fact that the BOSS models include only 25 feature wavelengths, respectively, suggesting that while redundant wavelengths were eliminated, some important features were also excluded during the optimisation process.

Further analysis of the characteristic wavelengths selected by the three methods indicates that all three feature selection strategies retained a relatively large number of characteristic wavelengths, leading to severe overfitting in the RBF neural network model. The IVSO-RBF model achieved an RMSEP value of 6.4609 and an $R_{P}^{2}$ value of 0.8659, representing the best predictive performance among the three models. However, compared to the CARS-PLS model, the RMSEP value of the IVSO-RBF model increased by 3.3397.

Table 5 lists the optimal PLS models based on the three variable selection methods. As shown in Table 4, compared to the full-spectrum PLS model, the optimised PLS models based on variable selection methods demonstrated improved detection accuracy and generalisation ability. Among them, the CARS-PLS model performed the best, achieving RMSEP and $R_{P}^{2}$ values of 3.1212 and 0.9687, respectively. When comparing the CARS-PLS model with the IVSO-RBF model, the CARS-PLS model exhibited superior predictive performance in this study. These results suggest that for the specific BHT-in-oil spectral dataset analysed in this study, the linear PLS model captured the correlation more effectively than the non-linear RBF model. While RBF networks have shown advantages in other complex food matrices, they appeared more susceptible to overfitting in this case, likely due to the limited sample size relative to the high-dimensional feature space.

It is worth noting that the optimal number of Latent Variables (LVs) in the PLS model was relatively high (LVs = 16). This can be attributed to the high complexity of the sample matrix, which involved nine different types and brands of edible oils. The model required more latent variables to account for the significant spectral variance introduced by the diverse fatty acid profiles and fluorescence backgrounds of these different oil matrices.

Regarding the data partitioning, a random splitting strategy was employed in this feasibility study to ensure that the calibration set covered the maximum diversity of both BHT concentrations and oil matrix types. While block cross-validation is often preferred for rigorous validation, the limited sample size (N = 126) combined with high matrix variability necessitated this approach to prevent underfitting the oil background features.

In summary, among the three algorithms used in this study for wavelength variable optimisation, CARS noticeably outperformed the others. The quantitative performance of the CARS-PLS model was also clearly superior to other models, demonstrating the feasibility of using Raman spectroscopy combined with the CARS-PLS model to detect BHT in edible oils. Although there is still room for improvement in the quantitative performance, this study provides a feasible method for detecting BHT in edible oils.

Compared with related studies, Wu et al. combined convolutional neural networks to detect antioxidants in peanut oil and achieved excellent results. However, their study was limited to detecting peanut oil among edible oils [34]. In contrast, this study utilised nine different types and brands of edible oils, introducing a certain degree of variability in each sample under every concentration gradient, which makes the model relatively more broadly applicable.

### 3.4. Limitations and Future Work

It is important to acknowledge the limitations of this study. First, the primary objective of this work was to evaluate the feasibility of using Raman spectroscopy combined with advanced variable selection algorithms (CARS, BOSS, IVSO) for the rapid screening of BHT. Therefore, a direct comparison with standard chromatographic methods (e.g., HPLC or GC-MS) was not performed in this specific study. While the chemometric models demonstrated high internal prediction accuracy, future work is strictly required to validate these spectroscopic results against standard reference methods to ensure regulatory compliance before industrial deployment. Second, the BHT concentration range tested (0.05–60 g/kg) was designed to validate model linearity and simulate gross adulteration, but it exceeds typical real-world regulatory levels (ppm range). Consequently, the model’s sensitivity for trace-level detection requires further optimisation. Third, this study utilised lab-spiked samples; future validation should involve commercial oil samples with endogenous BHT to account for potential matrix-analyte interaction differences. Additionally, the sample size (N = 126), while sufficient for a feasibility study, is relatively limited for testing broad model generalisation across different harvest years or processing conditions. Future work will focus on expanding the dataset and validating the method against standard chromatographic techniques under real-world production conditions. Moreover, future studies with larger datasets should consider block cross-validation strategies to further validate the model’s robustness against unseen concentrations.

## 4. Conclusions

The feasibility of using Raman spectroscopy combined with multivariate analysis for the rapid and accurate quantitative detection of BHT in edible oils was investigated in this paper. The important characteristic wavelength variables of Raman spectroscopy were selected based on three feature works: CARS, BOSS and IVSO. Subsequently, PLS and RBF detection models were developed using the optimally chosen variables. The results show that the PLS-based model is significantly better than the RBF-based model, and the detection accuracy of the PLS-based brother model is ranked as follows: CARS-PLS > BOSS-PLS > IVSO-PLS. Among these, the CARS-PLS model emerged as the optimal model, demonstrating that the CARS-PLS model has significant potential for the rapid determination of BHT content in edible oils. These findings demonstrate that the combination of Raman spectroscopy and chemometrics can serve as a promising tool for rapid screening of additives in edible fats. However, practical challenges remain in applying this method for low-level detection in regulatory contexts. Future research must address these challenges by improving spectral signal-to-noise ratios and validating the method’s robustness using extensive commercial datasets. This study represents a foundational step towards establishing a rapid, non-destructive screening protocol for food safety monitoring.

## Figures and Tables

**Table 1:** Types and brands of edible oil.

Number	Types	Brands
1	Corn oils	Knife (Lam Soon, Hong Kong, China)
2		Jinlongyu (Yihai Kerry Arawana Holdings Co., Ltd., Shanghai, China)
3		Luhua (Luhua Group Co., Ltd., Yantai, China)
4		Nissin (Nissin Foods Holdings Co., Ltd., Tokyo, Japan)
5	Soybean oils	Fulinmen (COFCO Corporation, Beijing, China)
6		Huifu (Sanhe Huifu Grain and Oil Group Co., Ltd., Sanhe, China)
7		Jinlongyu (Yihai Kerry Arawana Holdings Co., Ltd., Shanghai, China)
8	Sunflower oil	Fulinmen (COFCO Corporation, Beijing, China)
9	Peanut oil	Jinlongyu (Yihai Kerry Arawana Holdings Co., Ltd., Shanghai, China)

**Table 2:** Statistical results of BHT in edible oils for calibration and prediction sets.

Subsets	Number of Samples	Units	Maximum	Minimum	Mean	Standard Deviation
Calibration set	88	$g\cdot {kg}^{-1}$	60	0.05	9.664	17.155
Validation set	38	$g\cdot {kg}^{-1}$	60	0.05	12.967	17.879

**Table 3:** Statistical results of PLS models with different variable selection methods run 50 times.

Models	nVAR	RMSEP/g·kg^−1^		$R_{P}^{2}$		RPD
		Min–Max	Average ± SD	Min–Max	Average ± SD	Average
CARS	57 ± 26	3.1212–3.9670	3.5843 ± 0.2068	0.9494–0.9687	0.9586 ± 0.0048	4.99
BOSS	70 ± 22	3.2678–4.3348	3.7257 ± 0.2126	0.9396–0.9657	0.9553 ± 0.0051	4.80
IVSO	240 ± 133	4.3153–5.4689	5.0222 ± 0.0108	0.9039–0.9402	0.9186 ± 0.0108	3.56

**Table 4:** Comparison of optimal results of RBF models with different feature selection methods.

Models	nVAR	Parameter	RMSEC/g·kg^−1^	$R_{C}^{2}$	RMSEP/g·kg^−1^	$R_{P}^{2}$
BOSS-RBF	60	s = 100	0.0003	0.9999	8.5124	0.7672
CARS-RBF	46	s = 708	0.3838	0.9994	6.8380	0.8498
IVSO-RBF	82	s = 100	0.0003	0.9999	6.4609	0.8659

**Table 5:** Comparison of optimal results of PLS Models with different feature selection methods.

Models	nVAR	Parameter	RMSEC/g·kg^−1^	RMSECV/g·kg^−1^	$R_{C}^{2}$	RMSEP/g·kg^−1^	$R_{P}^{2}$	RPD
PLS	572	LV = 16	1.7698	2.5021	0.9892	4.8153	0.9255	3.71
BOSS-PLS	60	LV = 12	1.7172	2.1485	0.9898	3.2678	0.9656	5.47
CARS-PLS	44	LV = 13	1.6631	1.9560	0.9905	3.1211	0.9687	5.73
IVSO-PLS	212	LV = 14	2.4302	2.8644	0.9797	4.3153	0.9402	4.14

## Data Availability

The original contributions presented in the study are included in the article; further inquiries can be directed to the corresponding author.
